# Analysis of temperature behavior in biological tissue in photothermal therapy according to laser irradiation angle

**DOI:** 10.1080/21655979.2023.2252668

**Published:** 2023-09-03

**Authors:** Donghyuk Kim, Hyunjung Kim

**Affiliations:** Department of Mechanical Engineering, Ajou University, Suwon-si, Gyeonggi-do, Korea

**Keywords:** Apoptosis, gold nanoparticles, photothermal therapy, thermal damage, irradiation angle, squamous cell carcinoma

## Abstract

The type of death of biological tissue varies with temperature and is broadly classified as apoptosis and necrosis. A new treatment called photothermal therapy is being studied on this basis. Photothermal therapy is a treatment technique based on photothermal effects and has the advantage of not requiring incisions and, therefore, no bleeding. In this study, a numerical analysis of photothermal therapy for squamous cell carcinoma was performed. Photothermal agents used were gold nanoparticles, and the photothermal therapy effect was confirmed by changing the angle of the laser irradiating the tumor tissue. The effectiveness of photothermal therapy was quantitatively assessed on the basis of three apoptotic variables. Further, the volume fraction of gold nanoparticles in the tumor tissue and laser intensity with optimal therapeutic effect for different laser irradiation angles were studied. Thus, the findings of this study can aid the practical implementation of photothermal therapy in the future.

## GRAPHICAL ABSTRACT

**Figure:**
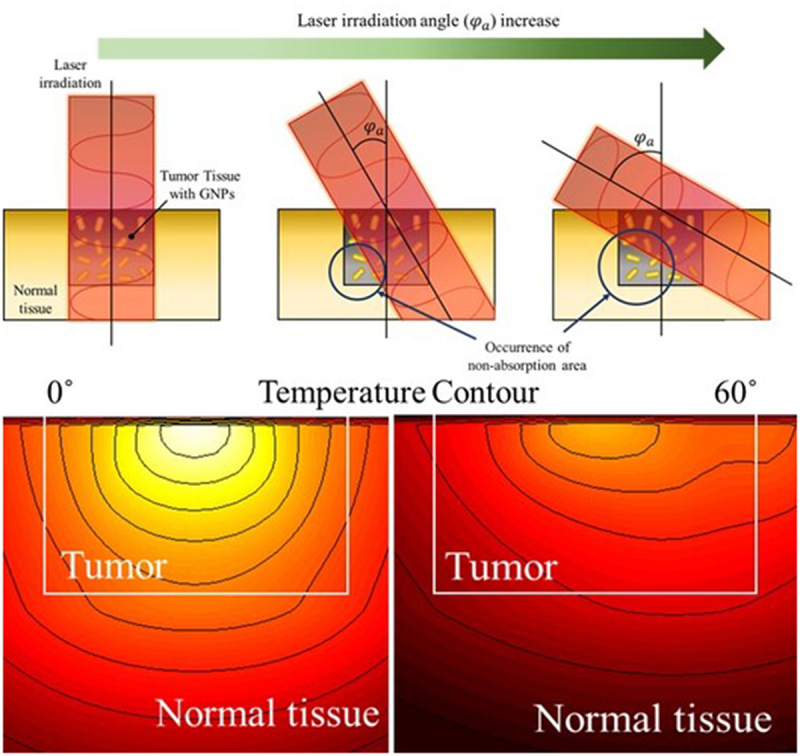

## 1. Introduction

Biological tissues undergo a variety of temperature-dependent behaviors [1,2]. Generally, tissue death begins when temperatures reach 43°C or higher and is expressed as apoptosis and necrosis, depending on the temperature range. Based on this phenomenon, a treatment technique known as photothermal therapy (PTT) is being researched in the medical field [3,4]. PTT is a treatment technique in which a laser, or light energy, is irradiated onto the affected area to kill tumor tissue by increasing the temperature. PTT has the advantage of not requiring an incision to perform the treatment, resulting in no bleeding and quick recovery [5,6].

As aforementioned, PTT aims to kill tumor tissue through increased temperature. In general, biological tissue dies in two forms, apoptosis and necrosis, depending on the temperature range. Apoptosis is known to occur between 43 and 50°C and refers to the phenomenon of self-destruction without affecting surrounding tissues. However, necrosis is known to occur at temperatures above 50°C and is the tissue death in the form of leakage of its contents. Maintaining the temperature range in which apoptosis occurs is a critical aspect of PTT, as any leakage of tumor tissue contents can lead to cancer metastasis to surrounding tissues [7,8].

A proper laser intensity and wavelength must be used to maintain the temperature range of apoptosis. Biological tissues have a high light absorption coefficient at visible wavelengths. Therefore, the thermal energy of the laser is absorbed by the tumor tissue and the surrounding normal tissue, causing unnecessary thermal damage. For this reason, PTT typically utilizes lasers in the near-infrared region. However, owing to the low light absorption coefficient of biological tissues in this region, using lasers in the near-infrared region alone has limitations in maintaining the temperature range where apoptosis occurs. Therefore, photothermal agents that increase the light absorption coefficient at a specific wavelength are injected into the tumor tissue to increase the light absorption coefficient [9–11]. Photothermal agents enhance light absorption at specific wavelengths through localized surface plasmon resonance (LSPR) and can be fabricated from various materials, including noble metals and polymers [12–15]. In practice, nanoparticles are made of various materials. It is broadly divided into noble metal nanoparticles and polymer nanoparticles [16,17]. Among the noble metal nanoparticles, gold nanoparticles are not toxic to the body and have been confirmed to be discharged from the body after injection. However, it has a relatively low photothermal conversion efficiency. On the other hand, polymer nanoparticles have a very high photothermal conversion efficiency depending on the combination, but their toxicity to humans has not yet been confirmed and they are not completely eliminated from the body.

In the field of heat transfer, research on PTT based on these considerations is being conducted. Capart et al. [18] performed a numerical study of PTT on a phantom that simulated a glioblastoma in a rat’s head. The temperature distribution on the rat head and glioblastoma under laser irradiation was calculated using thermal diffusion simulation and photoacoustic simulation, and the corresponding thermal damage was determined. In addition, the concentration of photo absorbers was locally varied at the tumor site to determine the therapeutic tendency with and without injections. Beik et al. [19] conducted a numerical analysis study of PTT based on computed tomography(CT) imaging. First, CT26 colon tumor-bearing mice were treated with alginate-coated AuNPs and then imaged with CT images. Information about the tumor geometry and distribution of the nanoparticles was then transferred to the simulation software for heat transfer modeling. The tumor temperatures predicted by the numerical simulations were confirmed to be in excellent agreement with the data measured in the in vivo experiments, suggesting that the developed model can be used to identify treatment conditions by adjusting various treatment parameters. Paul et al. [20] used finite element-based simulations to determine the temperature distribution of the tissue under laser irradiation, taking into account the cooling effect of blood vessels. A three-dimensional composite heat transfer equation for the tissue and blood regions was applied, and a laser heat term based on the Beer-Lambert law was applied to the energy equation. Biomimetic experiments were conducted to verify the temperature distribution under different conditions of blood vessels and laser irradiation to validate the numerical model. The parametric study confirmed that if the blood vessels are located at a depth of 3.5 mm or less from the surface, the temperature caused by laser heating is similar to that caused by the absence of blood vessels. It was also found that an increase in the tissue blood perfusion rate reduces the local cooling effect during laser heating. In addition, there are previous studies that have utilized various devices such as MRI and CT to obtain information(such as location, size) about the actual tumor tissue and conduct research on photothermal therapy [21,22].

To summarize, previous studies in the field of heat transfer have analyzed the temperature distribution in the medium through numerical simulations. In addition, the existence of thermal damage and the extent of thermal damage were analyzed through the Arrhenius thermal damage model. However, no quantitative information was provided on the temperature range maintenance for apoptosis, which is the core of PTT, and thermal damage to surrounding normal tissues was not analyzed at various temperature ranges. Lastly, previous studies have assumed that the laser is applied perpendicular to the tumor tissue. However, in actual treatment situations, it may not be possible to irradiate the laser perpendicularly to the patient because of various tumor locations and mechanical limitations. Therefore, this study investigated the temperature distribution in the medium under different laser irradiation angles. In addition, a quantitative analysis of how the laser irradiation angle affects the temperature maintenance of apoptosis in tumor tissue and thermal damage in normal tissues was performed on squamous cell carcinoma (SCC).

## 2. Materials and methods

### 2.1 Calculation of optical properties of nanoparticles and medium

This study entailed a numerical analysis of PTT using photothermal agents. Photothermal agents are substances that increase the light absorption coefficient at specific wavelengths by the LSPR phenomenon, which can compensate for the low light absorption coefficient of biological tissues in the near-infrared region. In general, photothermal agents have different light absorption coefficients for different wavelength ranges. Accordingly, optical properties at the target wavelength must be calculated. Various methods are used to calculate the optical properties of particles, such as Mie theory, finite difference time domain, and boundary element method; the discrete dipole approximation (DDA) method was used in this study [23]. The DDA method assumes that the dipoles are uniformly distributed inside the nanoparticle and then analyzes the interaction of each dipole to calculate the absorption and scattering efficiency of the particle.

First, the polarization vector *P* representing the dipole moment in unit volume must be calculated using Equation (1). Here, *α* and *E* are the polarizability and local electric field, respectively. The local electric field can be calculated using Equation (2), where *r*, *k*, and *A* represent the position vector, wavenumber, and interaction matrix between the dipoles, respectively. The interaction matrix *A* can be calculated as in Equation (3).

(1) $$P_{i}=\alpha_{i}\cdot E_{i}(r_{i})$$

(2) $$E_{i}(r_{i})=E_{0}e^{i(k\cdot r_{i})}-\sum_{i\neq j}^{N}A_{ij}\cdot P_{j}(i,j=1,2,3,\cdots ,N)$$

(3) $$\begin{array}{rl} & A_{ij}\cdot P_{j}\\ & \;\;\;\;\;\;\;\;\;\;\;\;=\frac{e^{i(k\cdot r_{ij})}}{r_{ij}^{3}}\left\{k^{2}r_{ij}\times (r_{ij}\times P_{j})+\frac{1-ikr_{ij}}{r_{ij}^{2}}\times \left[k^{2}P_{j}-3r_{ij}(r_{ij}\cdot P_{j})\right]\right\}(i\neq j) \end{array}$$

Finally, if *P* is calculated using the above equations, the optical cross-sectional area, *C*, can be calculated using that value, as shown in Equations (4) through (6). Here, the superscript * represents the compound conjugate symbol.

(4) Cabs=4πkE02∑i=1NIm[Pi⋅(αi−1)∗Pi∗]−23k3PiPi∗

(5) Cext=4πkE02∑i=1NIm(Einc,i∗⋅Pi)

(6) $$C_{sca}=C_{ext}-C_{abs}$$

Once the optical cross-sections are calculated, the absorption (*Q*_*abs*_), scattering (*Q*_*sca*_), and extinction efficiencies (*Q*_*ext*_) of the nanoparticle can be calculated, as shown in Equation (7). Here, *r*_*eff*_ and *V* denote the effective radius (Equation (8)) and volume of the nanoparticle, respectively.

(7) $$Q_{abs}=\frac{C_{abs}}{\pi r_{eff}^{2}}\;,\;Q_{ext}=\frac{C_{ext}}{\pi r_{eff}^{2}}\;,\;Q_{sca}=\frac{C_{sca}}{\pi r_{eff}^{2}}$$

(8) $$r_{eff}={\left(\frac{3V}{4\pi }\right)}^{1/3}$$

The optical coefficients of a nanoparticle can be calculated from the correlation equations proposed by Dombrovsky et al. [24], as shown in Equations (9) through (11). Here, *f*_*v*_ is the volume fraction of nanoparticles in the medium and *g* is the anisotropy factor, a dimensionless number that describes the distribution of light scattering.

(9) $$\mu_{abs,np}=0.75f_{v}\frac{Q_{abs}}{r_{eff}}\;,\;\mu_{sca,np}=0.75f_{v}\frac{Q_{sca}}{r_{eff}}$$

(10) $$\mu {{\;}^{\prime }}_{sca,np}=\mu_{sca,np}(1-g)$$

(11) $$\mu_{abs}=\mu_{abs,np}+\mu_{abs,m}\;,\;\;\mu {{\;}^{\prime }}_{sca}=\mu {{\;}^{\prime }}_{sca,np}+\mu {{\;}^{\prime }}_{sca,m}$$

### 2.2 Monte Carlo method and heat transfer model

This study used the Monte Carlo method to analyze the laser behavior inside biological tissues [25]. This technique simultaneously considers the degree of absorption and scattering due to the movement of one laser particle in the medium, and after repeatedly calculating the number of particles set at the beginning, the final light absorption distribution can be calculated through probability distribution analysis.

To calculate the light absorption distribution in the Monte Carlo method, it is necessary to calculate the azimuth(*ψ*) and deflection angle(*θ*), and the distance(*S*) traveled as one step progresses. First, the angle can be calculated from a random number (*ξ*) and an anisotropy factor (*g*) as shown in Equations (12) and (13).

(12) ψ=2πξ

(13) $$\cos\theta =\left\{\begin{array}{c} \frac{1}{2g}\left\{1+g^{2}-{\left[\frac{1-g^{2}}{1-g+2g\xi }\right]}^{2}\right\}if\;g>0\\ 2\xi -1\;if\;g=0 \end{array}\right.$$

(14) $$S=\frac{-\ln(\xi )}{\mu_{ext}}$$

Equation (14) is a formula for calculating the distance *S* traveled by a particle as it advances one step, which can be calculated from the ratio of a random number to the extinction coefficient of the medium (*μ*_*ext*_). Once the motion of a particle per step is determined, the proportion of energy it possesses after the movement decreases as shown in Equation (15). The energy proportion of the particle decreases by the ratio of the absorption(*μ*_*abs*_) and extinction coefficients(*μ*_*ext*_) of the medium, and it continues to move until its energy converges to zero.

(15) $$\Delta W=W\cdot \frac{\mu_{abs}}{\mu_{ext}}$$

(16) $$\phi_{z}[i_{z}]=\sum_{i_{r}=0}^{N_{r}-1}\phi_{rz}[i_{r},i_{z}]\cdot 2\pi (i_{r}+0.5)(\Delta r{)}^{2}$$

After repeating the process of Equations (12) to (15) with the number of particles initially set, the distribution of the absorbed heat in the medium can be calculated by probability distribution analysis as shown in Equation (16), where *ϕ*_*rz*_ and *ϕ*_*z*_ represent the absorbed photon probability density function and the energy density of photons in the depth direction, respectively, and *N*, *i*_*r*_, and *i*_*z*_ represent the initialized number of photons and the grid index in the r and z directions, respectively. Once the absorption distribution of the laser heat in the medium is calculated, the final temperature distribution in the medium can be calculated based on the heat diffusion equation including blood perfusion term in Equation (17). Here, *k*_*m*_, *ρ*, and *c*_*v*_ are the thermal conductivity, density, and specific heat, respectively, and *τ*, *q*, *q*_*perf*_ are the time, amount of heat absorbed by the medium, and blood perfusion term respectively. *q*_*perf*_ is calculated as in Equation (18), where *ω*_*b*_ represents tissue blood perfusion rate.

(17) $$\frac{\partial T}{\partial \tau }=\frac{q+\nabla \cdot (k_{m}\nabla T)+q_{perf}}{\rho c_{v}}$$

(18) $$q_{perf}=\rho_{b}\omega_{b}c_{b}(T_{b}-T)$$

### 2.3 Numerical model and conditions

**Figure 1.**
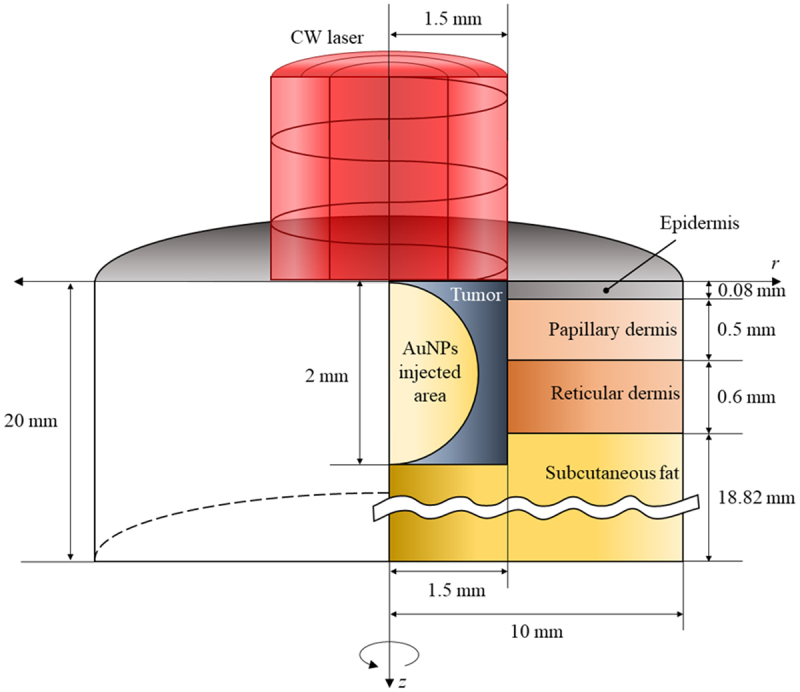
Schematic of numerical model.

**Figure 2.**
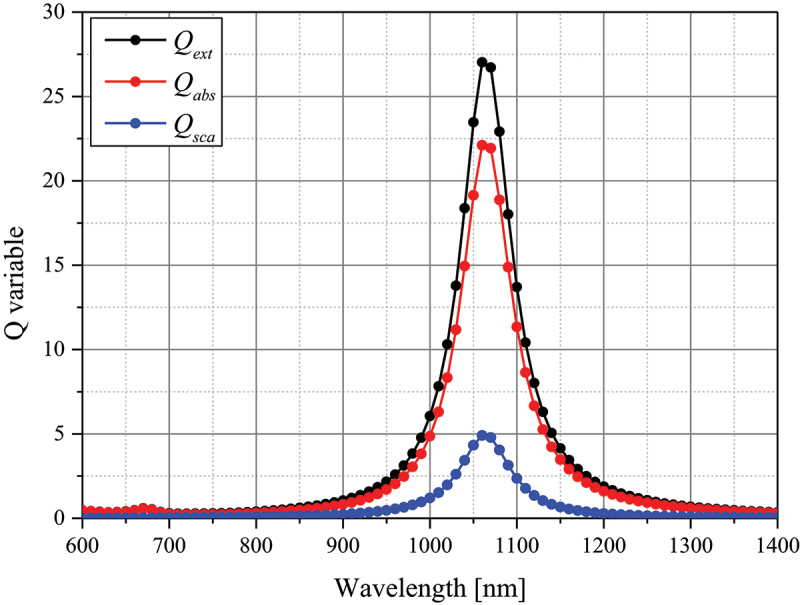
Spectral optical efficiency of gold nanoparticle (rod-type, *r*_*eff*_ = 20 nm).

In this study, numerical modeling of the occurrence of SCC inside the skin layer consisting of four stages was implemented. In case of tumor radius, it occurs in different sizes. The diameter of the SCC was set to 3 mm, as various studies showed that laser therapy was performed even when the diameter was 4 mm or less [26–28]. For the depth of SCC, it was set to a depth of 2 mm to simulate penetration into subcutaneous fat as shown in Figure 1. Normal tissue was set to 10 mm radius and 20 mm depth of cylindrical shape. Among the various photothermal agents, gold nanoparticles(AuNPs) were utilized and assumed to be injected in the form of a sphere with a radius of 1 mm in the center of the tumor tissue. The AuNPs used in the numerical analysis were rod-type nanoparticles with an effective radius of 20 nm and an aspect ratio of 6.67. The photothermal efficiency is maximized when the plasmon wavelength of the nanoparticles and the laser wavelength are matched. Therefore, to match the plasmon wavelength of the selected nanoparticles, a laser wavelength of 1064 nm was used in this study. The absorption, scattering, and extinction efficiencies of the rod-type gold nanoparticles used in this study at different wavelengths calculated through DDA method are shown in Figure 2.

Additionally, the laser wavelength of 1064 nm has sufficient penetration depth to allow the laser’s heat to penetrate a tumor tissue as deep as 2 mm. The irradiated laser utilized a 1064 nm continuous wave laser with a radius of 1.5 mm with a Gaussian distribution, and the irradiation time was fixed at 600 s. The thermal properties of the tumor tissue and each skin layer and the optical properties at the corresponding wavelengths are shown in Table 1.

**Table 1.** Properties of tumor tissue and each skin layers [29–36].

	t (mm)	k_*m*_ (W/mK)	$c_{v}$ (J/kgK)	ρ (kg/m^3^)	*ω*_*b*_ (1/s)	*μ*_*abs*_ (1/mm)	*μ*_*sca*_ (1/mm)	g
Tumor tissue	2	0.495	3421	1070	0.0063	0.047	0.883	0.8
Epidermis	0.08	0.235	3589	1200	0	0.4	45	0.8
Papillary dermis	0.5	0.445	3300	1200	0.0031	0.38	30	0.9
Reticular dermis	0.6	0.445	3300	1200	0.0031	0.48	25	0.8
Subcutaneous fat	18.82	0.19	2500	1000	0.0031	0.43	5	0.75

One of the main purposes of numerical analysis is to reduce the number of conditions or conditions that are difficult to perform in an experiment. Validation of the numerical modeling was performed in the authors’ previous study [37]. Biomimetic phantoms proposed by Surowiec et al. [38] and Iizuka et al. [39] were utilized; these phantoms are known to have similar thermal properties to human skeletal muscle tissue. Table 2 summarizes the composition of the phantom.

**Table 2.** Composition of phantom.

Composition	Combination ratio (weight %)	
Acrylamide	26	Acrylamide stock solution
Sodium chloride	1.05	
N,N’-methylenebisacrylamide	0.2	
DI water	71.7	
APS	1	Coagulant
TEMED	0.5	Catalyst

**Figure 3.**
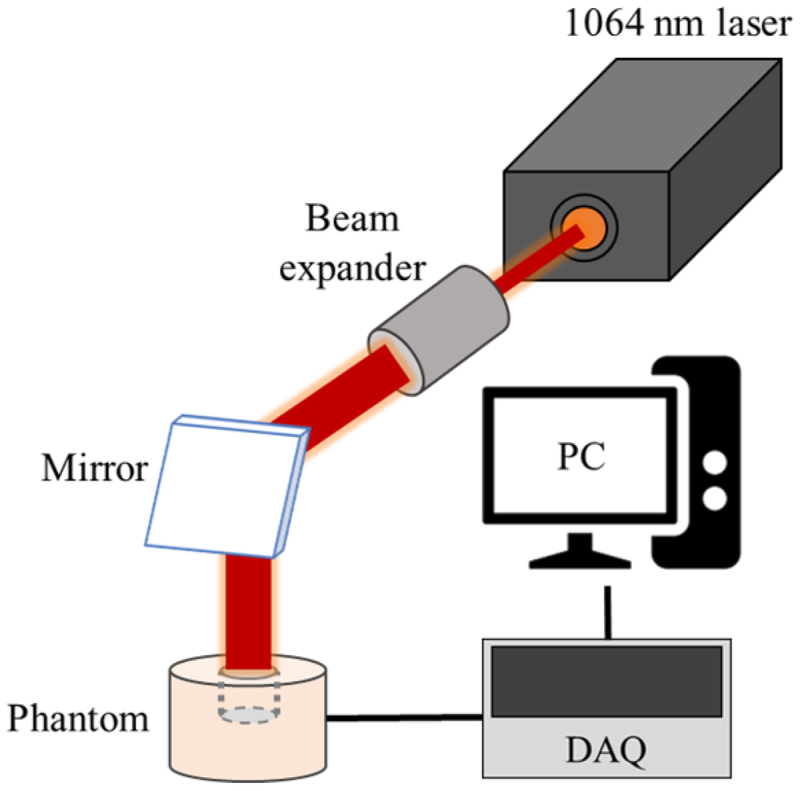
Schematic of phantom experiment setup.

**Figure 4.**
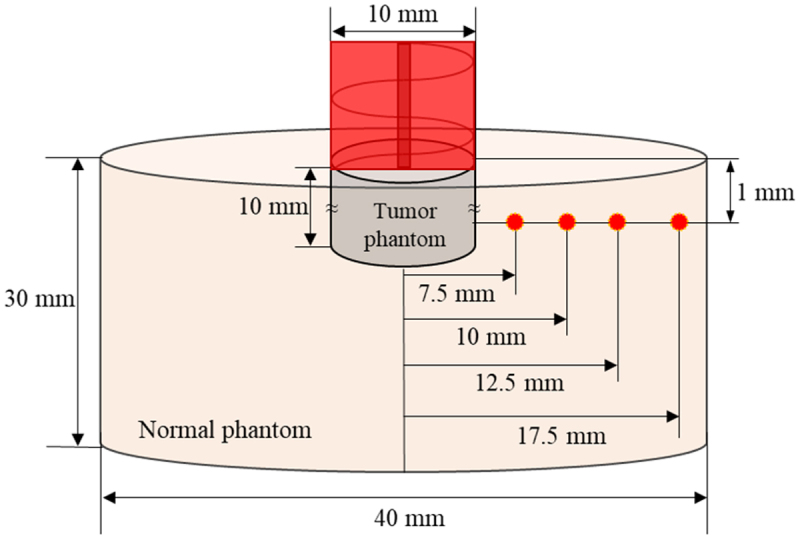
Temperature measurement point of phantom.

Figure 3 and 4 show a schematic of the phantom experiment and the thermocouple insertion locations. The phantoms were made to simulate normal tissue and tumor tissue injected with AuNPs and were irradiated with a laser at 1064 nm perpendicular to the phantom. The laser used was a Cobolt 04–01 series ‘Rumba’ model. The laser has a radius of 1 mm, which was extended to 10 mm with a beam expander, and the laser power is fixed at 0.4 W. The temperatures were measured at a total of four points in the radial direction over time. Upon comparing the numerical simulation and experimental results, the root mean square error was obtained as 0.1677 on average, thereby confirming the validity of the numerical simulation model. The phantom experiments used for verification were conducted with the laser irradiated vertically as mentioned above, and the results of changing the laser irradiation angle were verified through numerical analysis.

**Figure 5.**
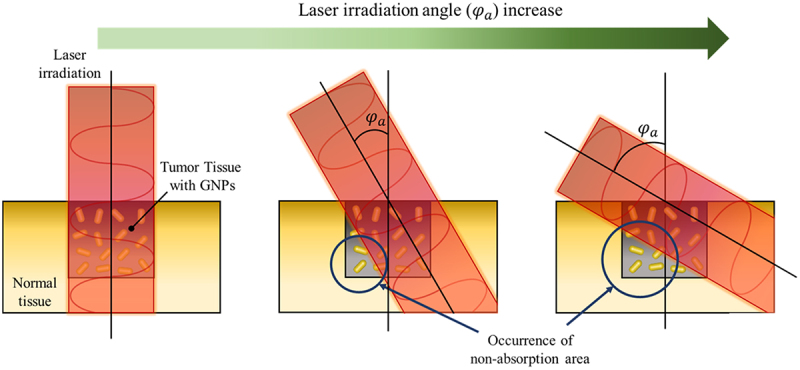
Change in the propagation path of the laser in the medium as the *φ*_*a*_ changes.

In this study, the temperature distribution inside the tissue was confirmed by changing the angle of the laser irradiated(*φ*_*a*_) to the tumor site as PTT was performed. Figure 5 depicts the change in the propagation path of the laser in the medium as *φ*_*a*_ changes. If the radius of the laser and the tumor tissue are the same, then all areas of the tumor tissue will be irradiated when the laser is irradiated vertically. However, if *φ*_*a*_ increases, the area inside the tumor that cannot be absorbed increases, as shown in the figure. This results in non-uniform heating within the tumor tissue, which prevents the tumor tissue from achieving the required temperature increase in the desired temperature range, and unnecessary thermal damage to the surrounding normal tissue due to the absorption of additional laser heat. However, in the actual treatment situation, the laser cannot be irradiated only in the vertical direction; therefore, it is necessary to analyze the effect of PTT according to various *φ*_*a*_ and identify the optimal treatment conditions at each *φ*_*a*_.

As aforementioned, this study aimed to analyze the effectiveness of PTT by changing *φ*_*a*_ of the laser irradiated to the tumor tissue. In addition, the temperature distribution in the medium was determined by changing the volume fraction of AuNPs(*f*_*v*_) in the tumor tissue and the intensity of the irradiated laser(*P*_*l*_), and the optimal conditions for PTT according to each *φ*_*a*_ were proposed. *φ*_*a*_ was set to six steps in 15° increments from 0° to 75° in the vertical direction, and *f*_*v*_ was set to four steps in 10^−1^ increments from 10^−3^ to 10^−6^. Since the optical properties of the entire medium change depending on the volume fraction of AuNPs injected, the absorption and reduced scattering coefficients are summarized in Table 3.

**Table 3.** Optical properties of tumor tissue with AuNPs.

Volume fraction of AuNPs (*f*_*v*_)	10^−3^	10^−4^	10^−5^	10^−6^
μabs (1/mm)	1881.163	188.158	18.858	1.928
μ’sca (1/mm)	79.619	40.604	4.855	1.280

In addition, *P*_*l*_ was set in 2 mW steps from 0 mW to 150 mW, and numerical analysis was performed for a total of 1,824 cases. Table 4 summarizes the variables and conditions for the parametric study.

**Table 4.** Parameters of numerical analysis.

Parameter	Case	Number	Remarks
Laser irradiation angle (*φ*_*a*_)	0 to 75°	6	Interval: 15°
Volume fraction of AuNPs (*f*_*v*_)	10^−3^ to 10^−6^	4	Interval: 10^−1^
Laser power (*P*_*l*_)	0 to 150 mW	76	Interval: 2 mW

## 3. Results

### 3.1 Light absorption and temperature distribution for varying laser irradiation angle

In this study, the temperature distribution in the medium was determined by varying the irradiated laser angle, changing the intensity of the laser and the volume fraction of the injected AuNPs according to each angle, among various conditions of photothermal therapy for SCCs occurring inside the skin layer. In addition, the calculated temperature distribution in the medium was used to quantitatively determine the treatment effect using the apoptotic variable proposed by Kim et al [37] to suggest optimal treatment conditions.

**Figure 6.**
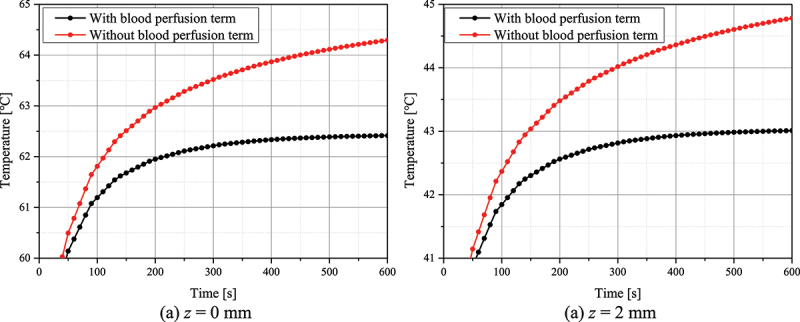
Temperature change with or without application of the blood perfusion term (*P*_*l*_=100 mW, *f*_*v*_=10^−4^).

In bioheat transfer, the application of blood perfusion terms is essential to calculate the temperature distribution in biological tissue. Figure 6 is the temperature comparison result over time when the blood perfusion term is applied and when it is not applied. In the case where *f*_*v*_ is 10^−4^ and the laser intensity is 100 mW in a situation where the laser is irradiated vertically, it shows the temperature over time at the surface (*z* = 0 mm) and 2 mm depth based on the center. As shown in the figure, it can be seen that the degree of temperature rise is different, and a difference of up to about 2 degrees occurs based on 600 seconds. Accordingly, the application of the blood perfusion term is essential for more accurate temperature calculation.

**Figure 7.**
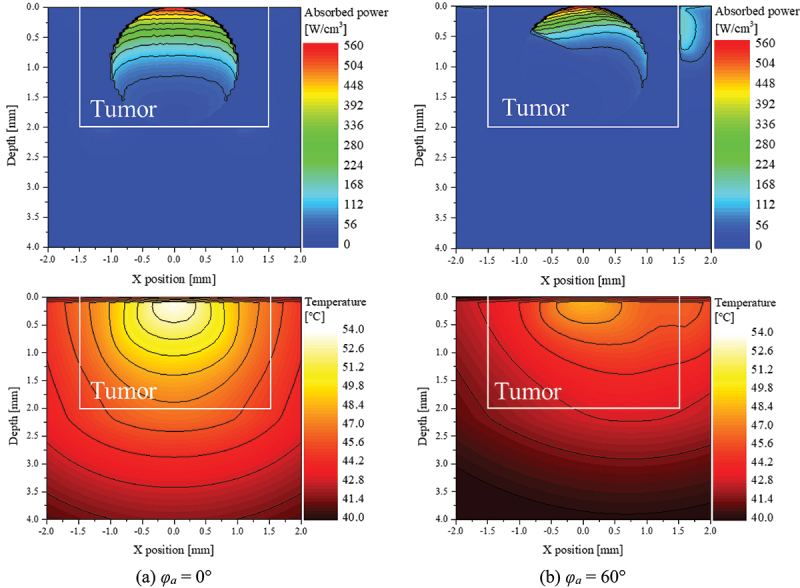
Light absorption and temperature distribution for various *φ*_*a*_ (*P*_*l*_=50 mW, *f*_*v*_=10^−6^).

Before calculating the apoptotic variable, which quantitatively identifies the effectiveness of PTT, the distribution of light absorption and temperature in the medium was confirmed. Figure 7 shows the light absorption in the medium and the temperature distribution at 600 s after laser irradiation when *φ*_*a*_ is 0° (Figure 7(a)), and 60° (Figure 7(b)) under the condition that *P*_*l*_ is 50 mW and *f*_*v*_ is 10^−6^. As shown in Figure 7(a), when the laser is irradiated perpendicularly to the tumor tissue, the light absorption occurs in the region where the AuNPs in the tumor are distributed, and the absorption is symmetrical about the center. As a result, the temperature in the medium also rises symmetrically and uniformly about the center. However, when *φ*_*a*_ is 60°, a region of non-absorption area occurs in the lower left corner, as shown in Figure 7(b). This causes uneven heating inside the tumor tissue, which means that the temperature rise is not uniform and only occurs in certain areas. Furthermore, light absorption in the right normal tissue occurred, causing an unnecessary temperature increase in the normal tissue. However, as thermal damage does not occur below 43°C, it is necessary to adjust the intensity of the laser appropriately to find a condition that maximizes the temperature inside the tumor tissue into the temperature range where apoptosis occurs while not causing thermal damage in normal tissue. On the basis of these considerations, this study calculated the light absorption and temperature distribution in all cases and applied it to the apoptotic variable to quantitatively analyze the effect of PTT to suggest the treatment condition with the optimal treatment effect at each *φ*_*a*_.

### 3.2 Maintaining the apoptotic temperature range within the tumor tissue

As aforementioned, biological tissues undergo diverse temperature-dependent death phenomena. Among them, apoptosis occurs between 43 and 50°C and is also called cell suicide because the tissue kills itself without affecting the surrounding tissue. In this study, the apoptosis retention ratio ($\theta_{A}^{*}$) proposed by Kim and Kim [37] was used to quantitatively determine the temperature at which apoptosis occurs in tumor tissue. $\theta_{A}^{*}$ can be defined as the average value of the volume ratio corresponding to the apoptosis temperature in the tumor tissue to the total volume of the tumor tissue over the entire treatment time. $\theta_{A}^{*}$ is a variable that verifies the results within the whole volume of the tumor tissue, which allows analyzing the temperature at which apoptosis occurs throughout the tumor tissue and indirectly confirms the effectiveness of the treatment. The maximum value of this variable is 1, which implies that the apoptosis temperature range is maintained at all points within tumor tissue for the entire treatment time.

**Figure 8.**
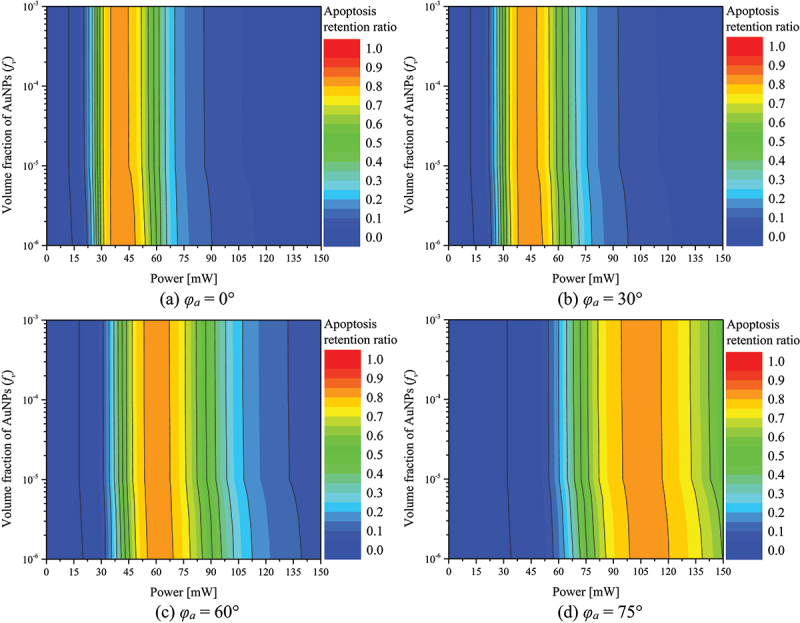
Apoptosis retention ratio($\theta_{A}^{*}$) for various *f*_*v*_ and *P*_*l*_.

Figure 8 shows $\theta_{A}^{*}$ with respect to *f*_*v*_ and *P*_*l*_ for different *φ*_*a*_. As shown in the figure, for each *φ*_*a*_, there exists a *P*_*l*_ value such that $\theta_{A}^{*}$ is maximized. It was identified that as *φ*_*a*_ increases, the *P*_*l*_ at which $\theta_{A}^{*}$ has maximized increases. This is because as *φ*_*a*_ increases, the amount of laser heat absorbed in the medium decreases, thereby necessitating a higher laser heat to increase $\theta_{A}^{*}$. In addition, in the case of *φ*_*a*_ below 60°, the maximum value of $\theta_{A}^{*}$ was obtained when *f*_*v*_ was 10^−6^, and in the case of 75°, the maximum value of $\theta_{A}^{*}$ was obtained when *f*_*v*_ was 10^−4^. The maximum value of $\theta_{A}^{*}$ at each *φ*_*a*_ and the treatment conditions are summarized in Table 5.

**Table 5.** Maximum value of $\theta_{A}^{*}$ and treatment conditions.

*φ*_*a*_ (°)	Treatment condition		Maximum $\theta_{A}^{*}$
	*f*_*v*_	*P*_*l*_ (mW)	
0	10^−6^	42	0.8487
15	10^−6^	42	0.8476
30	10^−6^	44	0.8435
45	10^−6^	50	0.8377
60	10^−6^	62	0.8329
75	10^−4^	106	0.8278

### 3.3 Quantitative analysis of thermal damage to surrounding normal tissue around tumor tissue

Thermal damage to surrounding normal tissue is inevitable when performing PTT. Since it is impossible to perform treatment without causing thermal damage to surrounding normal tissue, it is very important to minimize thermal damage. In this study, the thermal hazard retention value ($\theta_{H}^{*}$) was used to quantitatively analyze the amount of thermal damage to the surrounding normal tissue [37]. $\theta_{H}^{*}$ is a variable that helps analyze thermal damage by weighting different biological phenomena that occur in different temperature ranges and then summing the weights at each point. The range of normal tissue to be analyzed was selected from the tip of the tumor to a length equal to 50% of the length of the tumor tissue in order to quantify the amount of thermal damage to the normal tissue around the tumor tissue.

**Figure 9.**
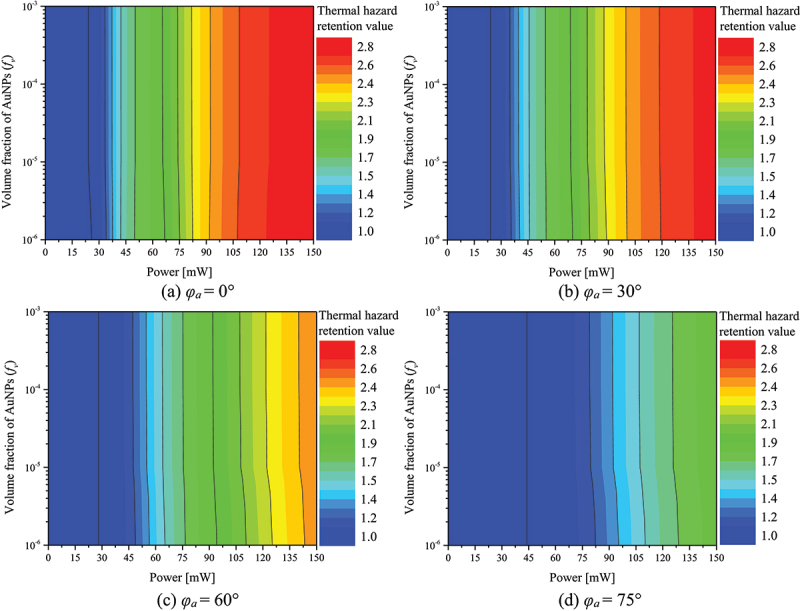
Thermal hazard retention value($\theta_{H}^{*}$) for various *f*_*v*_ and *P*_*l*_.

Figure 9 presents plots of $\theta_{H}^{*}$ with respect to *f*_*v*_ and *P*_*l*_ for different *φ*_*a*_. As shown in Figure 9, there is little difference in the thermal damage to normal tissue from *f*_*v*_ of 10^−3^ to 10^−5^. In general, in all the cases, it was observed that $\theta_{H}^{*}$ increases as *P*_*l*_ increases. This is because the laser heat is enhanced as *P*_*l*_ increases, absorbing more heat within the medium. If *φ*_*a*_ is 0°, all of the laser’s heat is absorbed by the tumor tissue, resulting in no heating of the surrounding normal tissue and only a temperature increase through conduction. However, owing to the presence of AuNPs, the heat generation in the tumor tissue is very high and a large amount of heat is transferred to the surrounding normal tissue. Moreover, as *φ*_*a*_ increases, the area of the tumor tissue absorbing the laser heat decreases, and the area of the surrounding normal tissue absorbing the heat increases, resulting in heat generation. However, the amount of heat transfer decreases because of the decrease in the heat absorption area of the tumor tissue. The surrounding normal tissue does not generate substantial heat because of the absence of AuNPs; therefore, the temperature does not increase significantly. Thus, it was confirmed that as *φ*_*a*_ increases, $\theta_{H}^{*}$ decreases at the same *P*_*l*_, and as *φ*_*a*_ increases linearly, $\theta_{H}^{*}$ decreases nonlinearly.

### 3.4 Confirmation of optimal conditions for various laser irradiation angle

Sections 3.2 and 3.3 detail the quantitative analyses of the maintenance of the apoptotic temperature range inside the tumor tissue and the thermal damage to the surrounding normal tissue, respectively. However, both are essentially simultaneous considerations when performing the treatment. Therefore, this study utilized an effective apoptosis retention ratio ($\theta_{eff}^{*}$), which can consider the above two points simultaneously [37]. This variable is defined as the ratio of $\theta_{A}^{*}$ to $\theta_{H}^{*}$, which was used to find the optimal treatment conditions that maximize the maintenance of apoptotic temperature inside the tumor tissue while minimizing thermal damage to the surrounding normal tissue.

**Figure 10.**
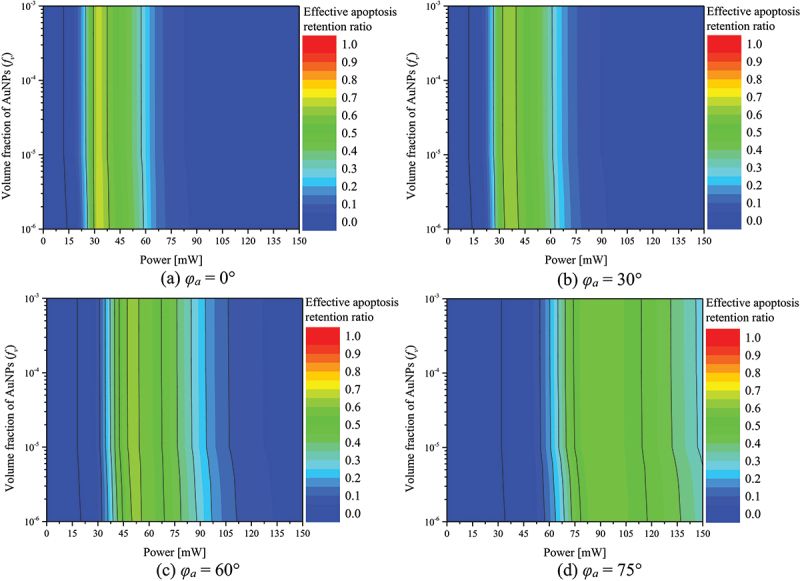
Effective apoptosis retention ratio($\theta_{eff}^{*}$) for various *f*_*v*_ and *P*_*l*_.

Figure 10 shows $\theta_{eff}^{*}$ with respect to *f*_*v*_ and *P*_*l*_ for different *φ*_*a*_. As with $\theta_{A}^{*}$, there is a condition under which $\theta_{eff}^{*}$ is maximized for all cases. For *φ*_*a*_ below 15°, the maximum value of $\theta_{eff}^{*}$ was obtained when *f*_*v*_ is 10^−6^. However, after 30°, the maximum value of $\theta_{eff}^{*}$ was obtained when *f*_*v*_ is 10^−3^. This is because for *φ*_*a*_ of 15° or less, most of the laser heat is absorbed within the tumor tissue and there is less absorption from the surrounding normal tissue. Therefore, it is more beneficial from a temperature increase perspective to have a deeper and wider range of laser heat absorption by lowering the *f*_*v*_. However, when *φ*_*a*_ exceeds 30°, the area at the bottom of the tumor tissue that does not directly absorb the laser heat increases, and the adjacent normal tissue directly absorbs the laser heat. Therefore, it is more beneficial to reduce the laser penetration depth by increasing *f*_*v*_ to absorb the laser heat at the surface from the viewpoint of the temperature rise of the tumor tissue. Furthermore, it was observed that $\theta_{eff}^{*}$ has a lower *P*_*l*_ with a maximum compared to the results for $\theta_{A}^{*}$. This is because thermal damage to the surrounding normal tissue occurs more in *P*_*l*_, which maintains the apoptosis temperature range in the tumor tissue to the maximum. Therefore, it is more beneficial from a therapeutic point of view to reduce $\theta_{H}^{*}$, even if it means losing $\theta_{A}^{*}$. *f*_*v*_ and *P*_*l*_ values at which the treatment effect is maximized at each *φ*_*a*_, and the $\theta_{eff}^{*}$ at that time, are summarized in Table 6.

**Table 6.** Optimal photothermal therapy conditions for various *φ*_*a.*_

*φ*_*a*_ (°)	Optimal condition		
	*f*_*v*_	*P*_*l*_ (mW)	$\theta_{eff}^{*}$
0	10^−6^	32	0.6723
15	10^−6^	34	0.6609
30	10^−3^	34	0.6396
45	10^−3^	40	0.6281
60	10^−3^	50	0.6138
75	10^−3^	86	0.5863

**Figure 11.**
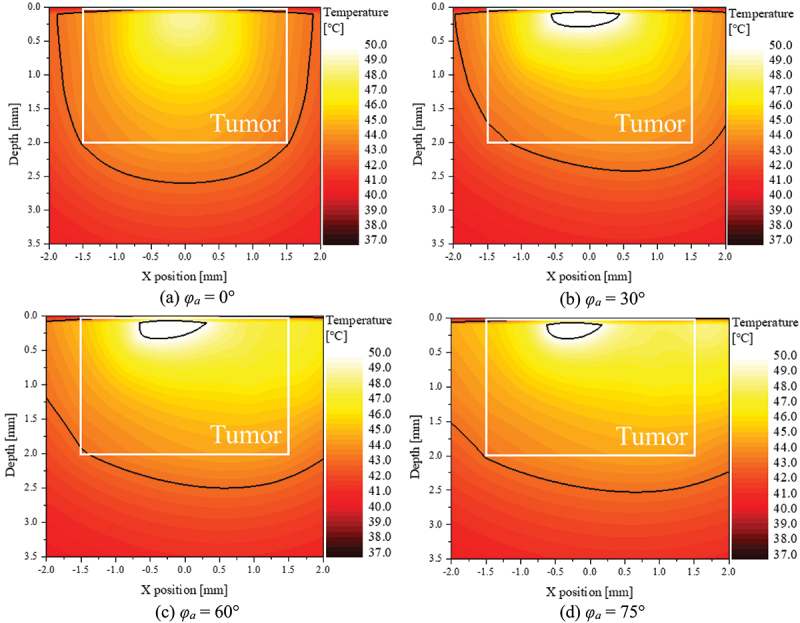
Temperature contour for optimal conditions for various *φ*_*a*_.

Lastly, to verify the results of $\theta_{eff}^{*}$, temperature distribution at the point *y* = 0 was confirmed. Figure 11 shows the temperature contour in the *XZ* plane under optimal treatment conditions for each laser irradiation angle. This result shows the temperature distribution at a laser irradiation time of 600 seconds. In the graph, the area within the white box represents the tumor tissue area, and the area within the black line represents the area between 43 and 50°C, the apoptosis temperature range. First, the intratumoral temperature distribution under optimal treatment conditions at all laser irradiation angles shows that most of the region corresponds to the apoptosis temperature range. This is because at least 82% of the region falls into the apoptosis temperature band, as confirmed by the results for $\theta_{A}^{*}$. On the other hand, under optimal conditions, the temperature of the surrounding normal tissue is also in the apoptosis temperature range, indicating that thermal damage is occurring. This is confirmed by the fact that under optimal conditions, the result of $\theta_{eff}^{*}$ is lower than $\theta_{A}^{*}$. As the laser irradiation angle increases, the area corresponding to the apoptosis temperature band in the surrounding normal tissue increases, resulting in a lower $\theta_{eff}^{*}$.

## 4. Discussion

This study quantitatively confirmed the treatment effects of various laser irradiation angles on skin cancer PTT for SCC and determined optimal treatment conditions. The study entailed numerical analyses and was based on the assumption that SCC has developed inside a skin layer consisting of four stages. The distribution of light absorption by the laser in the medium was calculated using the Monte Carlo method, and the optical properties of the gold nanoparticles were calculated using the DDA method.

Finally, the apoptosis retention ratio, which identifies the degree of retention of the apoptosis temperature range in the tumor tissue; the thermal hazard retention value, which quantitatively calculates the amount of thermal damage to the surrounding normal tissue; and the effective apoptosis retention ratio, which simultaneously identifies the above two factors, were used to propose the conditions for optimal treatment effects for each laser irradiation angle. The findings are expected to accelerate the implementation of PTT as they afford the effects of various laser irradiation angles in actual PTT situations. However, in actual practice, the shape of the tumor will be very irregular, different from the shape of the tumor presented in this study. In a practical situation, it seems that it is essential to first identify the shape of the tumor using devices such as MRI and CT, and depending on the shape, the actual amount and location of the gold nanoparticles and the laser radius and intensity should be set differently. In addition, it seems to be very important from a therapeutic point of view to have a collaboration between optics, biology, and heat transfer in the future to be able to map the location of the tumor in real time and present temperature mapping as the treatment is performed.

## 5. Conclusion

In conclusion, treatment effects of various laser irradiation angles on skin cancer PTT was quantitatively confirmed by numerical analysis using apoptotic variable. As the laser irradiation angle increased, it was found that non-absorption area of laser heat occurred at the bottom of the tumor, resulting in a decrease in the range corresponding to the intratumoral apoptosis temperature range. In addition, thermal damage occurs due to the increase in the laser absorption area of the surrounding normal tissue, so the treatment should be performed by setting the appropriate laser intensity and volume fraction of AuNPs according to each irradiation angle.

## Nomenclature

*C*: cross-section area ($m^{2}$)
*c*_*v*_: Specific heat (J/kgK)
*E*: Electric field (N/C)
*F*: Fluence rate ($1/m^{2}s$)
*g*: Anisotropy factor
*i*: index of grid element
*k*: wavenumber of radiation (1/m)
*k*_*m*_: Thermal conductivity (W/mK)
*N*: number of photons
*P*: Polarization vector ($C/m^{2}$)
*P*_*l*_: Intensity of laser (W)
*q*: Volumetric heat source ($W/m^{3}$)
*Q*: Dimensionless efficiency factor
*r*: Position vector
*r*_*eff*_: Effective radius of nanoparticle (m)
*S*: Photon’s moving distance per 1 step (m)
*t*: Thickness (m)
*T*: Temperature (K)
*W*: Energy weight of photon (J)
***Greek symbols*** 
α
polarizability ($C^{2}m^{2}/J$)
θ
Deflection angle (${\;}^{\circ }$)
$\theta_{A}^{*}$
Apoptosis retention ratio
$\theta_{eff}^{*}$
Effective apoptosis retention ratio
$\theta_{H}^{*}$
Thermal hazard retention value
μ
Optical coefficient (1/m)
ξ
Random number
ρ
Density ($kg/m^{3}$)
$\phi_{z}$
Energy density of photon
$\phi_{rz}$
Absorbed photon probability density
τ
Time (s)
ψ
Azimuth (${\;}^{\circ }$)
$\varphi_{ir}$
Injected radius ratio of AuNPs
*ω*_*b*_: Blood perfusion rate (1/s)
***Subscripts*** 
*abs*: Absorption
*ext*: extinction
*m*: Medium
*np*: Nano particle
*sca*: Scattering
*x, y, z*: Notation of direction
***Superscripts*** 
**+**: Next element
−: Previous element

## Abbreviations

DDA, discrete dipole approximation; LSPR, localized surface plasmon resonance; PTT, photothermal therapy; SCC, squamous cell carcinoma
